# AI-doscopist: a real-time deep-learning-based algorithm for localising polyps in colonoscopy videos with edge computing devices

**DOI:** 10.1038/s41746-020-0281-z

**Published:** 2020-05-18

**Authors:** Carmen C. Y. Poon, Yuqi Jiang, Ruikai Zhang, Winnie W. Y. Lo, Maggie S. H. Cheung, Ruoxi Yu, Yali Zheng, John C. T. Wong, Qing Liu, Sunny H. Wong, Tony W. C. Mak, James Y. W. Lau

**Affiliations:** 1 Division of Biomedical Engineering Research, Department of Surgery, The Chinese University of Hong Kong, Hong Kong SAR, People’s Republic of China; 2 Division of Vascular and General Surgery, Department of Surgery, Prince of Wales Hospital, The Chinese University of Hong Kong, Hong Kong SAR, People’s Republic of China; 3 College of Health Science and Environmental Engineering, Shenzhen Technology University, Shenzhen, People’s Republic of China; 4 Division of Gastroenterology and Hepatology, Department of Medicine and Therapeutics, Institute of Digestive Disease, The Chinese University of Hong Kong, Hong Kong SAR, People’s Republic of China; 5 Department of Electrical and Electronic Engineering, Xi’an Jiaotong-Liverpool University, Suzhou, People’s Republic of China; 6 Division of Colorectal Surgery, Department of Surgery, The Chinese University of Hong Kong, Hong Kong SAR, People’s Republic of China

**Keywords:** Cancer, Translational research

## Abstract

We have designed a deep-learning model, an “Artificial Intelligent Endoscopist (a.k.a. AI-doscopist)”, to localise colonic neoplasia during colonoscopy. This study aims to evaluate the agreement between endoscopists and AI-doscopist for colorectal neoplasm localisation. AI-doscopist was pre-trained by 1.2 million non-medical images and fine-tuned by 291,090 colonoscopy and non-medical images. The colonoscopy images were obtained from six databases, where the colonoscopy images were classified into 13 categories and the polyps’ locations were marked image-by-image by the smallest bounding boxes. Seven categories of non-medical images, which were believed to share some common features with colorectal polyps, were downloaded from an online search engine. Written informed consent were obtained from 144 patients who underwent colonoscopy and their full colonoscopy videos were prospectively recorded for evaluation. A total of 128 suspicious lesions were resected or biopsied for histological confirmation. When evaluated image-by-image on the 144 full colonoscopies, the specificity of AI-doscopist was 93.3%. AI-doscopist were able to localise 124 out of 128 polyps (polyp-based sensitivity = 96.9%). Furthermore, after reviewing the suspected regions highlighted by AI-doscopist in a 102-patient cohort, an endoscopist has high confidence in recognizing four missed polyps in three patients who were not diagnosed with any lesion during their original colonoscopies. In summary, AI-doscopist can localise 96.9% of the polyps resected by the endoscopists. If AI-doscopist were to be used in real-time, it can potentially assist endoscopists in detecting one more patient with polyp in every 20–33 colonoscopies.

## Introduction

**Fig. 1:**
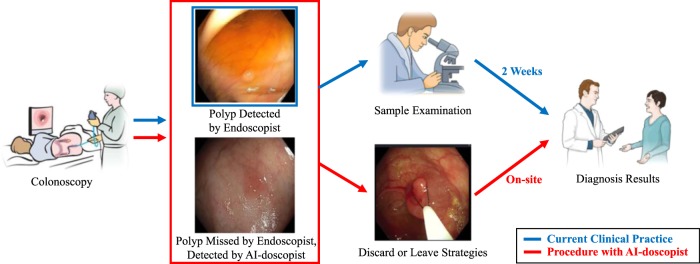
An illustration of the future use of AI-doscopist, a.k.a. “Artificial Intelligent Endoscopist”, during colonoscopy. Colonoscopy can effectively reduce CRC incidence and mortality, but is contingent on a high-quality examination. Polyps that are diminutive in size (<5 mm), sessile in type and flat in shape are more frequently being missed during colonoscopy. To maximize the quality of colonoscopy, an automated tool is designed to assist endoscopists by highlighting regions of a possible polyp during colonoscopy.

Colorectal cancer (CRC) is top three commonest cancers worldwide, with an estimated 1.8 million new diagnoses and 881 thousand deaths occurred in 2018^1^. Colonoscopy can effectively reduce CRC incidence and mortality, but is contingent on a high-quality examination. Polyps that are diminutive in size (<5 mm), sessile in type and flat in shape are more frequently being missed during colonoscopy^2^. Human factors such as visual fatigue and inadvertent overlook were also found to be contributing to the missed lesions. For example, one study showed that polyp detection rates decline over time during an endoscopist’s working day by ~4.6% per hour^3^. An automated tool can assist endoscopists by highlighting a region of a possible polyp during colonoscopy, thus maximizing the quality of colonoscopy, as illustrated in Fig. 1.

Although computer-aided detection methods for polyp detection have been actively studied in the past, most of them were based on hand-crafted feature engineering methods^4,5^. These methods require strong domain knowledge and are less robust to background noises. The advantage of the hand-crafted features is that the predictions are easier to be explained. Some of these methods can even achieve near real-time performance (at 10 frames per seconds, fps)^6^. On the other hand, the recent explosion of data opens up new opportunities for applying deep-learning models for a range of computing tasks. Deep convolutional neural networks (CNNs) required large amount of data for training; however, with sufficient training, deep features can be stored in the model and used to classify or detect different objects. The models can achieve promising results even if the same class of objects possess very different features^7^. Therefore, deep-learning models have been shown to be useful in different tasks in both non-medical^7^ and medical domains^8^, including classification of diminutive colorectal polyps^9,10^.

Based on our previous work on using deep-learning models to detect and localise colorectal lesions in colonoscopy videos^11^, we aim to evaluate in this study the agreement between endoscopists and the AI-doscopist (Artificial Intelligent Endoscopist), a deep-learning-based computer-aided model we developed for colorectal lesion localisation.

## Results

### Results from image-based analysis

**Fig. 2:**
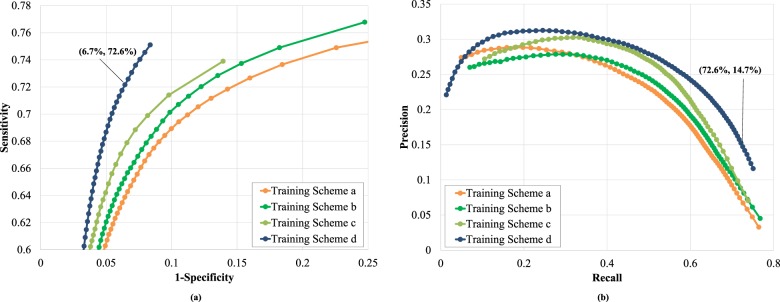
The image-based performance of AI-doscopist on Dataset B under different training schemes. **a** the Receiver Operating Characteristic curves and **b** the Precision–Recall curves. In Training Scheme a, AI-doscopist learnt only the spatial features from a random subset of 33,819 original colonoscopy images. In Scheme b, the training set was enlarged to a random subset of 119,703 original colonoscopy and non-medical images. In Scheme c, AI-doscopist learnt both the spatial and temporal features from a random subset of 119,703 original colonoscopy and non-medical images. In Training Scheme d, the spatial and temporal features were learnt from a larger, random subset of 191,493 colonoscopy and non-medical images. A total of 34,469 images were used for validation in each case.

We evaluated the proposed model on different platforms. When the input image resolution was fixed at 608 × 608, the model ran at around 28 frames per second (fps) on a Nvidia GTX 1080Ti and at 37 fps on a Nvidia GTX 2080Ti. Figure 2a, b presents the Receiver Operating Characteristic (ROC) curves and the Precision–Recall (PR) curves for AI-doscopist on the testing dataset under different training schemes, respectively. The model trained using Scheme d (threshold = 0.1) was selected based on its performance in the validation dataset and used for further analysis. The selected model achieved an image-based sensitivity of 72.6% and specificity of 93.3% when evaluated on Dataset B. The accuracy and precision of it were 92.0% and 14.7%, respectively.

Table 1 shows the evaluation performance of AI-doscopist on different testing datasets, using training scheme d and the selected threshold 0.1.

**Table 1:** Image-based evaluation results of AI-doscopist using training scheme d.

Dataset	No. of polyp images	No. of non-lesion images	True-positives	False-negatives	True-negatives	False-positives	Image-based sensitivity	Image-based specificity
Dataset A	4313	13,261	3106	1207	12,880	480	72.0%	97.1%
Dataset B	65,958	3,603,892	47,877	18,082	3,363,076	277,407	72.6%	93.3%
Dataset B.1	65,958	N/A	47,877	18,082	N/A	N/A	72.6%	N/A
Dataset B.2	N/A	69,157	N/A	N/A	72,238	3514	N/A	95.7%

### Results from Polyp-based analysis

**Fig. 3:**
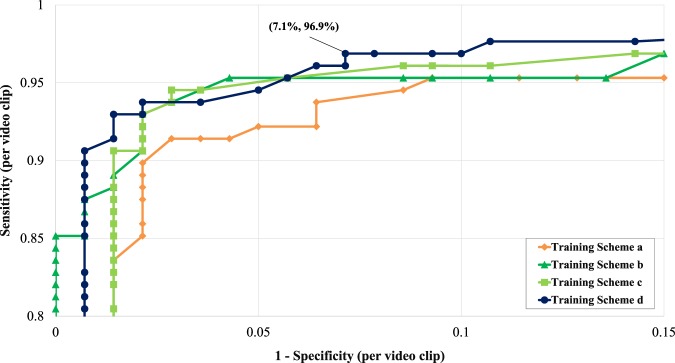
The polyp-based performance of AI-doscopist on Datasets B.1 and B.2 under different training schemes. Although Training Schemes b, c, and d resulted in significant different performances in the image-based analysis (as shown in Fig. 2), their performances are comparable in the polyp-based analysis.

Figure 3 shows the polyp-based evaluation of AI-doscopist under different training schemes. On average, AI-doscopist correctly localised a polyp for 15.0 out of 20.6 s. For video clips without a polyp, AI-doscopist falsely detected in 1.0 out of 20.6 s. AI-doscopist correctly localised 124 out of 128 polyps (polyp-based sensitivity = 96.9%) when *n* = 16% (i.e. a polyp was correctly localised in at least 16% of the frames of a video clip). If the same criteria were used to evaluate 140 video clips randomly selected from 70 patients, who had no lesions detected, AI-doscopist made 10 out of 140 false detections (polyp-based specificity = 92.9%). On average, 147.2 frames (5.9 s) were falsely detected in each of these 10 video clips.

### Estimation of potential increase in polyp detection rate (PDR)

PDR is defined as the number of patients found with at least one polyp divided by the total number of patients who underwent colonoscopy. For Dataset C, the endoscopists had found at least one polyp in 62 patients (total number of polyps = 130). No polyp was found in 40 patients and their full colonoscopies were screened by AI-doscopist off-line after colonoscopy. The regions highlighted by AI-doscopist were then reviewed by an endoscopist for a second time. The endoscopist confirmed with high confidence that four regions highlighted by AI-doscopist in three patients were possible polyps. Another four regions were confirmed with low confidence in another two patients as possible polyps. Therefore, if AI-doscopist were to be used in real-time, the estimated possible increase in PDR is around 3–5%, as summarised in Table 2.

**Table 2:** Estimated increase in polyp detection rate based on the evaluation on Dataset C.

	1st time diagnosis by endoscopist during colonoscopy	2nd time reviewed by an endoscopist, after screened by AI-doscopist	
		With high confidence	With high or low confidence
No. of patients diagnosed with a polyp	62	65 (=62 + 3)	67 (=62 + 5)
No. of patients without any lesion detected	40	37 (=40 − 3)	35 (=40 − 5)
No. of polyps detected	130	134 (=130 + 4)	138 (=130 + 8)
Polyp detection rate	60.8%	63.7%	65.7%

## Discussion

Using deep learning in endoscopy has been gaining interest in the research communities^12^. Compared to previous studies in this area, our study contributed uniquely in the following aspects: In this study, we explicitly trained our model using data obtained from multiple databases collected from different regions in the world, including colonoscopy and non-medical databases collected by our own research group. Different training schemes have been proposed and tested on the same dataset, which include over 3.71 million images from the full colonoscopy videos of 144 patients, and labelled by information obtained from 144 endoscopy reports and 70 pathology reports. No images/videos were preselected manually for testing. Rather, the full colonoscopy videos were evaluated for image-based and polyp-based analysis. Moreover, the training and testing datasets in our study were obtained from completely different patients. Therefore, we found that the evaluation of our model is extremely close to reality, providing solid evidence to carry out prospective study of AI-doscopist in real clinical setting. Since our method was trained on images from around the world, it is robust to different endoscopy setting, scopes, and instruments.

Although a number of studies have been conducted in this area, the evaluation methods, datasets, and metrics varied from study to study. As a result, comparison between different studies is not straight forward. Most studies trained and evaluated their methods on preselected still images and are not comparable to our study objectives and design. Two recent publications evaluated computer-aided diagnosis algorithms in full colonoscopy or colonoscopy video clips^13,14^. One publication presented an algorithm developed based on SegNet, which after being trained and tested on their own colonoscopy images and videos, can achieve over 90% in both image-based sensitivity and specificity^13^. The same model achieved a sensitivity of 88% when tested on a public database (CVC-ClinicDB)^13^. Another publication presented the evaluation of a system for detecting, rather than localising, polyps in colonoscopy achieved an image-based sensitivity and specificity of 90.0 and 63.3%, respectively^14^. It detected 94% (47 out of 50) polyps, but also resulted in 60% false-positive detection in 85 short non-lesion video clips. Their results suggested that one must observe for a tendency of over-diagnosing in artificial intelligent systems.

Our proposed algorithm correctly localised 124 out of 128 polyps (polyp-based sensitivity = 96.9%) and missed four polyps (Fig. 3). It resulted in only 7.1% false detections in short video clips (10 out of 140), which is considerably lower than previous work^14^. Our evaluation method demonstrated that AI-doscopist can correctly localise most of the polyps; however, it cannot localise the same polyp in every frame. This is consistent with the general knowledge of endoscopists, who often need to orbit around a suspicious lesion before they can make judgement. Furthermore, we have also included an estimation of the potential improvement in PDR if AI-doscopist were to be used back-to-back with conventional colonoscopy. Based on our evaluation on Dataset C, we postulated that there can be a 3–5% increase in PDR. That is, AI-doscopist can possibly help endoscopists to detect one more patient with polyp in every 20–33 colonoscopies. This is given that endoscopists are confident enough to resect polyps missed by AI-doscopist. This remains to be verified in future study.

Although the precision of AI-doscopist seems to be relatively low (<0.3), one should take into account that in the full colonoscopies, the images without a polyp normally outnumber those with a polyp (≈56:1). The correct predictions were made in 47,877 out of 65,958 (72.6%) polyp images; but only 277,407 false predictions were made in 3,776,900 regions without a lesion (7.3%). The image-based specificity for the evaluation on Datasets A, B, and B.2 were 97.1, 93.3, and 95.7%, respectively (Table 1). The polyp-based specificity for the evaluation on Dataset B.2 was 92.9% (=100 − 7.1%, Fig. 3). The image-based analysis suggested that the model was detecting one suspicious object in every second (for 25 fps). Nevertheless, the polyp-based analysis suggested that when one considered short video clips of 20 s, only 7.1% of these video clips have detected an object for more than 3.2 s (=20.6 × 16%). Therefore, to confirm whether a polyp has been detected by AI-doscopist, the endoscopist can orbit around a suspicious region for at least 3 s (up to 15–20 s) during colonoscopy to reduce false-positives. Furthermore, “false positives” in this study include (1) missed polyps; (2) hyperplastic or other polyps, which were detected but not resected; and (3) polyps/resected polyps localized during polypectomy or removal from the colon, during which we did not label the images due to limited manpower. Therefore, it is expected that the true precision and specificity will be higher if AI-doscopist were to be run in real-time during colonoscopy.

Moreover, we labelled our gold standard frame-by-frame by rewinding the videos from the start of biopsy of a polyp to the first appearance of a polyp. Note that this is a very tough criterion compared to other previous studies, which typically asked multiple endoscopists to confirm the existence of polyps in each endoscopic image. When labelling the gold standard in our study, some videos were played forward and backward multiple times before the labelling can be confirmed. It is suspected that if each endoscopic image were independently reviewed by an endoscopist, some of the polyps may not be accurately located in the blurry frames of the video clips. To our best knowledge, most of the previous papers did not report whether the gold standard was labelled in frames that are recorded during motion or out of focus. This is suspected to be one of the major reasons causing the differences in the reported performance metrics between our study and previous studies.

It is necessary to standardise the evaluation scheme for different computer-aided diagnosis systems in this area. Setting an evaluation guideline will help end-user to select the best system. In this study, we presented the definition of TP, TN, FP, FN, polyp-based sensitivity, and image-based specificity in the **Evaluation Metrics** Section. Note that some studies in the engineering domains defined image-based specificity as TN/(TN + FP)^15^, while a number of recent studies defined image-based specificity as TN/(Total Number of Non-lesion Images)^13,14^. The former definitions will result in a lower specificity if multiple regions were wrongly identified from the same frame, whereas the later definition do not take into account multiple false detections in the same frame. We adopted the later definition in this study since we found that this definition better shows the user experience of an endoscopist in reality.

In summary, we presented the image-based and polyp-based evaluation results of a real-time artificial intelligent algorithm for localising polyps in colonoscopy videos, using different medical and non-medical datasets for training. We tested AI-doscopist on the full colonoscopies of 144 patients. AI-doscopist correctly localised 124 out of 128 polyps (polyp-based sensitivity = 96.9%), missed four polyps, and achieved an image-based specificity of 93.3%. If AI-doscopist were to be used as a second observer during colonoscopy, it can potentially help endoscopists to detect one more patient with polyp in every 20–33 colonoscopies. Benefits of the use of AI-doscopist in improving adenoma detection rate, compared with other related techniques such as Endocuff, need to be verified in future prospective studies.

## Methods

### Algorithm description

**Fig. 4:**
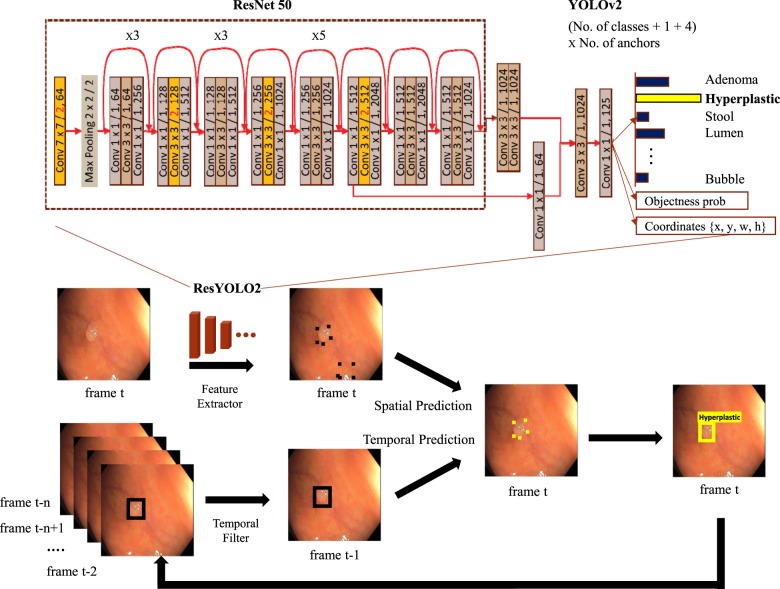
An overview of the algorithm design of AI-doscopist. AI-doscopist was constructed based on ResNet50, YOLOv2, and a temporal tracker. The model was found to perform reasonably well with a good trade-off between speed and accuracy in earlier studies. The feature extractor was adopted from a modified version of ResNet50. A one-stage object detector, YOLOv2, was selected for localising objects in each image in real-time. Predicted boxes that were unlikely polyp were removed and overlapped predicted boxes were combined using the non-maximum suppression method. Temporal information was incorporated by using the majority votes of the prediction results within a sliding window.

AI-doscopist was constructed based on one of our earlier works^11^, which was built from ResNet50^16^, YOLOv2^17^, and a temporal tracking algorithm. The model was found to perform reasonably well with a good trade-off between speed and accuracy. As shown in Fig. 4, AI-doscopist adopted ResNet50 as the feature extractor^16^. ResNet50 was constructed by 16 residual blocks, each consisted of three convolutional layers with different channel widths and strides. We modified the ResNet50 architecture by changing the channel width of the last convolutional layer and by adding two convolutional layers. Furthermore, we added a routing layer to retain the high resolution feature maps for concatenation. On the other hand, YOLOv2^17^ is a one-stage object detection system targeted for real-time processing. It divided the input image into a certain number of grids and predicted the confidence and the location of an object in each grid using a single regression-based CNN structure. The dimension of the output layer of the combined structure was determined by the number of girds, the number of classes, and the number of predefined anchors. YOLOv2 was found to be useful for the current application since a polyp can appear in different spatial location in an image. Prediction boxes that were unlikely polyp were removed and overlapped prediction boxes were combined using the non-maximum suppression method. Temporal information was incorporated by using the majority votes of the prediction results within a sliding window, which was six consecutive frames in length.

The backbone network of AI-doscopist was first pre-trained with 1.2 million non-medical images collected from the public online database ImageNet^18^. Additional learning on a training dataset for 90 epochs used stochastic gradient descent with a learning rate of 0.001, weight decay of 0.0005 and momentum of 0.9. All learned weights were monitored by the validation dataset to avoid overfitting. The learned weights that gave the highest sensitivity, given the specificity was over 0.9, when evaluated on the validation dataset were selected as the final model for testing.

### Training and validation datasets

The training and validation datasets to fine-tune AI-doscopist consisted of colonoscopy and non-medical images. The images were obtained from seven databases around the world, including four public online colonoscopy databases, two private databases formed by colonoscopy images/videos from two local hospitals, and one non-medical database. Table 3 summarises the number of images in each of the 7 databases: (1) CVC-ColonDB^19^, (2) CVC-ClinicDB^20^, (3) ETIS-LaribDB^21^, (4) AsuMayoDB^22^, (5) CU-ColonDB^9^, (6) ACP-ColonDB_530_, and (7) Selected Google Images. Details of the first five databases have been described in our previous studies^9,11,23^. As most of the images in the previous five databases consisted of images with polyps, we constructed the sixth database from videos of colonoscopies collected from our Endoscopy Centre. To construct this database, written informed consents were obtained from patients before colonoscopy during June to October 2017. Excluding 19 patients with abnormality found but no biopsy taken, 133 patients with corrupted/missed videos, and 14 patients whose lesion cannot be labelled, 364 patients were included in this database, namely ACP-ColonDB_530_. Data from 220 patients were used for training and validation (ACP-ColonDB_530-Train_), while data from 144 patients (68.0 ± 8.8 years old and 69 males) were used for testing (ACP-ColonDB_530-Test_). A total of 110 h of colonoscopy videos were recorded from 364 patients by seven endoscopists. The objects found in the colonoscopy images were classified into 13 categories, namely “Adenomatous Polyp”, “Hyperplastic Polyp”, “Other Polyp”, “Bleeding”, “Lumen”, “IC Valve”, “Normal Colon Structure”, “Instrument”, “Stool”, “Bubble”, “Artefact”, “Inside Colon Background”, and “Outside Colon Background”.

**Table 3:** Summary of the number of images used for training and validating AI-doscopist.

Name of database	Training subset		Validation subset	
	No. of polyp images	No. of non-lesion images	No. of polyp images	No. of non-lesion images
CVC-ColonDB	297	N/A	82	N/A
CVC-ClinicDB	485	N/A	127	N/A
ETISDB	150	N/A	46	N/A
AsuMayoDB_Train_	3237	1842	619	55
CU-ColonDB	634	N/A	164	N/A
ACP-ColonDB_530_	72,350	116,250	13,973	19,403
Selected Google Images	N/A	2893	N/A	N/A
Total (before augmentation)	77,153	120,985	15,011	19,458
Total (after augmentation)	160,618	130,472	N/A	N/A

The total length of the colonoscopy videos we collected for the training dataset were 57 h. We included images with a polyp as much as possible (72,350 images). In order to maintain a relatively balanced ratio between images with and without a polyp, we randomly selected 116,250 images without a polyp for training. Most of these images were selected based on running the training dataset with an earlier version of AI-doscopist. “False Positives” were manually checked and re-labelled to other categories. “False Negatives” were confirmed and other non-polyp labels that can possibly affect the localization of the polyp were added in the same image. “True Negatives” were randomly selected for inclusion for training. The selection ratio is around 3.7%, which is a trade-off between acceptable performance, labelling efforts and time required for training.

In addition, 2893 non-medical images were obtained from Google for training. AI-doscopist simultaneously with the colonoscopy images. These images were found to share common features as colorectal polyps and therefore we hypothesized that training AI-doscopist with these images can improve the polyp localisation performance. Specifically, the images were searched online using keywords that described a polyp. Objects included were “blood vessels”, “fingers”, “skin”, “eggs”, “nuts”, “red meats”, …, and “tomatoes.” The images were broadly classified into seven categories, namely, “Cell”, “Food”, “Body”, “Nature”, “Plant”, “Pattern”, and “Others”.

As summarised in Table 3, the images were divided into the training and validation subsets. The ratio of colonoscopy images used for training to validation was around 6:1. In particular, from ACP-ColonDB_530_, 182 patients (160 polyps) and another 38 patients (32 polyps) were used for training and validation, respectively. The training subset was further augmented by random rotation (0°, 90°, 180°,and 270°), flipping (horizontal and vertical), Gaussian smoothing (sigma ranged from 0.5 to 2), or different combinations of these operations. The number of images with polyps were increased from 77,153 to 160,618, and those without a polyp were increased from 120,985 to 130,472. Four training schemes were used: (a) when only spatial features were learnt from a random subset of 33,819 original colonoscopy images; (b) when only spatial features were learnt from a random subset of 119,703 original colonoscopy and non-medical images; (c) when both spatial and temporal features were learnt from a random subset of 119,703 original colonoscopy and non-medical images; and (d) when both spatial and temporal features were learnt from a random subset of 191,493 colonoscopy and non-medical images. A total of 34,469 images were used for validation in each case.

This study and the recording of the endoscopic videos were approved by the Clinical Trial Ethics Committee of The Chinese University of Hong Kong (CREC 2017.064). Written informed consent were obtained from 144 patients who underwent colonoscopy and their full colonoscopy videos were prospectively recorded for evaluation.

### Study protocol

After pre-training and fine-tuning AI-doscopist, we evaluated its performance on a public database (Dataset A), as well as 144 full colonoscopies (Dataset B). Furthermore, a private database consisted of 102 full colonoscopy videos (Dataset C) was used to estimate the potential increase in PDR if AI-doscopist were to be used in real-time screening.

To compare the performance of AI-doscopist with existing algorithms, we first evaluated it on a public online database, AsuMayoDB (Dataset A). AsuMayoDB was originally used for the MICCAI endoscopic vision challenge in 2015 and a number of algorithms have reported their performance using this database. In this study, we chose to evaluate AI-doscopist on AsuMayoDB such that a direct comparison with existing algorithms can be made. Besides the 20 videos used for training and validating the algorithm, 18 short colonoscopy video clips from AsuMayoDB has been designated for algorithm testing^22^. Nine videos have one polyp each and the rest have no polyps. A total of 4313 polyp images and 13261 non-lesion images were extracted from the 18 videos for evaluation in this study. Each frame in the videos has a respective reference image marked with a binary mask. Black region in the reference image indicates non-lesion region. On the contrary, white region represents the polyp area. The reference images were initially created by Arizona State University.

As aforementioned, data from 144 patients of ACP-ColonDB_530-Test_ were used for evaluation (Dataset B). Their colonoscopy videos were recorded in MP4 format at 25 fps. Resected tissues were sent for histological diagnosis and used as the gold standard. Among them, 128 polyps were found in 70 patients. According to the histology analysis, the 128 polyps were 110 adenomatous, 10 hyperplastic, and 8 mucosal polyps. Adenomatous polyps contributed to 85.9% in this test dataset. No polyp was found in 74 patients.

**Fig. 5:**
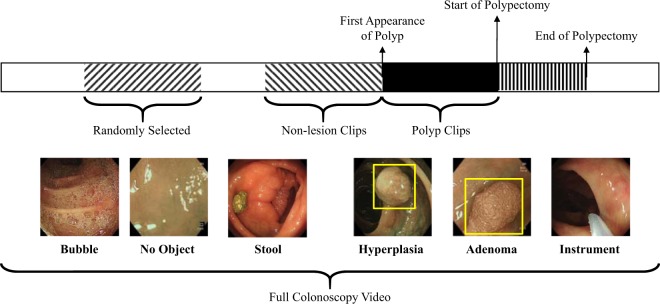
An illustration of video clips and types of images that were found in a full colonoscopy video. Three timepoints were marked for each full colonoscopy video: (1) the first appearance of the polyp, (2) the start of the biopsy/polypectomy procedure, confirmed by the first appearance of an endoscopic tool; and (3) the end of its biopsy/polypectomy procedure. Nil were recorded when no polyp was found in a colonoscopy video. The colonoscopy images were screened by an earlier version of AI-doscopist. All localised objects were classified into 13 categories, namely “Adenomatous Polyp”, “Hyperplastic Polyp”, “Other Polyp”, “Bleeding”, “Lumen”, “IC Valve”, “Normal Colon Structure”, “Instrument”, “Stool”, “Bubble”, “Artefact”, “Inside Colon Background”, and “Outside Colon Background”.

As shown in Fig. 5, three timepoints were marked for each full colonoscopy video collected: (1) the first appearance of the polyp, (2) the start of the biopsy/polypectomy procedure, confirmed by the first appearance of an endoscopic tool; and (3) the end of its biopsy/polypectomy procedure.

Two subsets were generated from Dataset B. Dataset B.1 contained 128 short video clips, each started with the first appearance of a polyp, and ended with the beginning of the polypectomy of that polyp. Dataset B.2 consisted of 140 short video clips extracted from 70 patients without any detected polyp. The average duration of the video clips in Dataset B.2 was 20.6 s, which is equivalent to the average duration of the polyp video clips of Dataset B.1. Figure 5 illustrates the type of images found in Datasets B, B.1 and B.2 from the 144-patient cohort.

To estimate the potential increase of PDR with AI-doscopist compared to traditional colonoscopy, an endoscopist was invited to re-examine a subset of highlighted colonoscopy video clips (Dataset C). Dataset C consisted of a 102-patient cohort who underwent colonoscopy from June to July 2017. In this cohort of patients, 62 patients had one or more polypectomies, while 40 had no biopsies taken during their procedures. Videos of the 40 patients who had no biopsies taken were screened by AI-doscopist for potentially missed polyps. The predictions of AI-doscopist were transformed into bounding boxes to highlight suspicious regions and overlaid on the original full colonoscopy. Videos clips with highlighted regions were segmented and re-examined by an endoscopist. The protocol is similar to performing a back-to-back colonoscopy. The endoscopist was invited to comment whether the region highlighted by AI-doscopist correctly identified a polyp, together with his level of confidence (high or low).

### Gold standard labelling

Dataset A is an online database which the gold standard of each image has been provided by a binary mask. For Dataset B, the polyp areas were marked image-by-image with a bounding box in each polyp clip. In order to efficiently and accurately label each image in the dataset, each video clip was first screened using one of our previously developed polyp detection algorithms^11^. The gold standard was then confirmed by fine-tuning the bounding box in each image manually.

### Evaluation metrics for image-based analysis

The prediction generated from AI-doscopist was in the form of a 6-element vector that indicated the class (either a polyp or not), confidence level, centre coordinates, width and height of the detected object, respectively. Only the predicted bounding boxes for the three polyp classes were evaluated in this study. The image-based metrics used to measure the correctness of each predicted bounding box were as follows:

1. True-positive (TP) counts the number of polyp areas, which has at least one prediction box with the centre point fallen within the area marked by the ground truth. If the centroids of multiple predicted boxes fall inside the same ground-truth bounding box, it will only be counted as one TP.
2. False-positive (FP) counts in any image the number of prediction boxes fallen outside the ground-truth polyp area.
3. True-negative (TN) counts the number of non-lesion images that have no prediction boxes.
4. False-negative (FN) counts the number of polyp areas where none of the centroids of the predicted boxes fall within the area marked by the ground truth.

In addition, the image-based sensitivity, specificity, precision, and accuracy were calculated using the following set of equations:

$$\begin{array}{*{20}{l}} {{\mathrm{Image}} - {\mathrm{based}}\,{\mathrm{Sensitivity}} = {\mathrm{TP/}}\left( {{\mathrm{TP}} + {\mathrm{FN}}} \right);} \hfill \\ {{\mathrm{Image}} - {\mathrm{based}}\,{\mathrm{Specificity}} = {\mathrm{TN}}/{\mathrm{Total}}\,{\mathrm{Number}}\,{\mathrm{of}}\,{\mathrm{Non - lesion}}\,{\mathrm{Images}};} \hfill \\ \begin{array}{l}{\mathrm{Precision}} = {\mathrm{TP}}/\left( {{\mathrm{TP}} + {\mathrm{FP}}} \right);{\mathrm{and}}\\ {\mathrm{Accuracy = }}\left( {{\mathrm{TP}} + {\mathrm{TN}}} \right)/\left( {{\mathrm{TP}} + {\mathrm{TN}} + {\mathrm{FP}} + {\mathrm{FN}}} \right).\end{array} \hfill \end{array}$$

The ROC curves and the PRC were plotted for different training methods of AI-doscopist. Both ROC curves were made by varying the algorithm threshold from 0.01 to 1.0 in steps of 0.01. The confusion matrix of the predictions was calculated for the selected model.

### Evaluation metrics for polyp-based analysis

Furthermore, we analysed the number of polyps that were missed by AI-doscopist. AI-doscopist was considered as correctly localising a polyp if it made prediction in at least n% of the frames of a short video clip, and the ROC curves for n ranging from 9 to 44% were plotted. The polyp-based sensitivity is calculated as the number of detected polyps over the total number of polyp clips. The polyp-based specificity is calculated as the number of falsely detected objects over the total number of non-polyp clips.

### Reporting summary

Further information on research design is available in the Nature Research Reporting Summary linked to this article.

## Supplementary information

Reporting Summary

## Data Availability

Data are available on request due to privacy or other restrictions.

## Code availability

The codes are available upon request. Users are required to accept a license agreement before using the codes.
